# Ningxiang Pig-Derived *Parabacteroides distasonis* HNAU0205 Alleviates ETEC-Induced Intestinal Apoptosis, Oxidative Damage, and Inflammation in Piglets

**DOI:** 10.3390/ani14152156

**Published:** 2024-07-24

**Authors:** Zichen Wu, Longlin Zhang, Hongkun Li, Junyao Li, Zihao Zhang, Bie Tan, Jing Wang

**Affiliations:** 1 Hunan Provincial Key Laboratory for the Products Quality Regulation of Livestock and Poultry, College of Animal Science and Technology, Hunan Agricultural University, Changsha 410128, China; zichen123@stu.hunau.edu.cn (Z.W.); zllwithann@gmail.com (L.Z.); lhko328@163.com (H.L.); 15054348532@stu.hunau.edu.cn (J.L.); zzbs0399@stu.hunau.edu.cn (Z.Z.); 2 Yuelushan Laboratory, Changsha 410128, China

**Keywords:** *Parabacteroides distasonis*, Ningxiang pig, intestinal apoptosis, oxidative damage, inflammation, SCFAs, weaned piglets

## Abstract

**Simple Summary:**

Weaning stress poses significant physiological challenges to piglets, often manifesting as intestinal disturbances, such as inflammation and impaired intestinal function, which ultimately affect growth and health. Probiotics can alleviate these adverse effects of stress and promote healthy growth in piglets. *Parabacteroides distasonis* (PBd), as a potential probiotic, has shown efficacy in preventing various diseases, but its anti-diarrheal effects in weaned piglets remain unclear. This study aims to investigate the effects of PBd derived from Ningxiang pigs on growth performance, intestinal apoptosis, oxidative damage, and inflammation in *Enterotoxigenic Escherichia coli* (ETEC)-challenged weaned piglets. The results show that oral administration of PBd can improve intestinal health and attenuate ETEC-induced intestinal damage in piglets.

**Abstract:**

Weaning is a critical stage in the growth and development of piglets, often inducing stress reactions. This study aims to investigate the effects of *Parabacteroides distasonis* (PBd) derived from Ningxiang pigs on growth performance, intestinal apoptosis, oxidative damage, and inflammation in ETEC-challenged weaned piglets. A total of 22 Duroc × Landrace × Yorkshire (DLY) piglets, 24 days old with similar body weights, were randomly divided into three groups: Control (*n* = 7), ETEC (*n* = 7), and PBd + ETEC (*n* = 8). The results show that, compared to the Control group, ETEC challenge led to decreased growth performance, reduced villus height in the duodenum and jejunum, increased crypt depth in the duodenum, a decreased villus-height-to-crypt-depth ratio, increased expression of apoptosis-related genes (*Caspase-8* and *Caspase-9*), increased expression of oxidative damage-related genes (*Nrf2*, *GSH-PX*, *mTOR*, and *Beclin1*), increased expression of inflammation-related genes (*Myd88*, *P65*, *TNF-α*, and *IL-6*), and reduced the contents of SCFAs in the colonic chyme (acetate, propionate, butyrate, valerate, and total SCFAs). Compared to the ETEC group, the PBd + ETEC group alleviated the reduction in growth performance, mitigated intestinal morphological damage, and reduced the expression of the aforementioned apoptosis, oxidative damage, and inflammation-related genes with the increase in SCFAs. In conclusion, PBd derived from Ningxiang pigs effectively reduces ETEC-induced intestinal damage in weaned piglets, improves intestinal health, and increases the content of SCFAs in the colonic chyme, thereby enhancing growth performance.

## 1. Introduction

Weaning is a critical stage in the growth and development of piglets [1,2]. Early weaning can induce stress reactions, leading to imbalances in antioxidant function, immunosuppression, reduced disease resistance, increased morbidity and mortality, and decreased growth performance, which collectively result in significant economic losses to the farming industry [1]. Enterotoxigenic *Escherichia coli* (ETEC) is one of the main pathogens that destroy the intestinal function of animals, and it is a globally recognized pathogenic bacterium that causes severe diarrhea in piglets [3]. ETEC infection can induce intestinal oxidative stress [4] and inflammatory response [1] in piglets, resulting in intestinal damage and growth retardation [3].Thus, exploring effective measures to alleviate weaning stress in piglets is of great significance for healthy breeding and the high-quality development of pig farming.

Several studies have demonstrated that probiotics can mitigate the adverse effects of indigestion, diarrhea, and growth stagnation caused by weaning stress in piglets, thereby promoting their growth and development [5,6,7]. *Parabacteroides distasonis* (PBd), a potential probiotic known for producing secondary bile acids, is commonly found in the gastrointestinal tract of humans and animals [8,9]. Previous research has shown that PBd can offer protective benefits against conditions such as colitis, obesity, and non-alcoholic fatty liver disease (NAFLD) [10,11,12,13]. Wang et al. demonstrated PBd can alleviate obesity and metabolic dysfunction by producing secondary bile acids and succinic acid [10]. Kuang et al. reported that PBd significantly alleviated hepatic fat accumulation in mice, and its metabolite γ-linolenic acid can inhibit the expression of hepatic CYP7A1 by activating the peroxisome proliferator-activated receptor alpha (PPAR-α) signaling pathway, thereby improving NAFLD [11], while PBd and its other metabolite pentadecanoic acid also effectively inhibited the progress of Non-alcoholic steatohepatitis [12]. In addition, multiple researchers have identified that PBd has the anti-inflammatory and anti-tumorigenic presence of intestinal tumors, especially in colorectal carcinogenesis [13]. Ma et al. reported that PBd can significantly alleviate DSS (dextran sulfate sodium)-induced ulcerative colitis and reduce colonic mucosal damage in mice [14]. Gaifem J et al. showed that PBd could improve the intestinal epithelial barrier function of colitis-susceptible mice and plays a synergistic protective effect with *Akkermansia muciniphila* [15]. In addition, Cuffearo B et al. showed that five strains of PBd (which were isolated from the gut microbiota of adults and neonates) showed anti-inflammatory effects, and some strains could significantly restore the expression of occludin and proliferation of Treg cells, regulate the body’s immunity, and improve intestinal inflammation [16]. PBd has shown anti-inflammatory and immunomodulatory effects in many studies. However, it remains unclear whether PBd can enhance resistance to ETEC infection in weaned piglets.

In this study, we isolated PBd from the feces of healthy Ningxiang pigs and utilized the ETEC piglet diarrhea model to investigate the effect of PBd derived from Ningxiang pigs on growth performance, intestinal apoptosis, oxidative damage, and inflammation in weaned piglets. The aim is to provide theoretical references for improving pig health and advancing the development of next-generation microecological preparations.

## 2. Materials and Methods

The animal experimental protocols were conducted according to the Institutional Animal Care and Use Committee of Hunan Agriculture University (Changsha, Hunan, China).

### 2.1. Piglet Experimental Design

The *Parabacteroides distasonis* HNAU0205 (PBd) strain used in this study was initially isolated from the feces of healthy suckling Ningxiang male piglets, and the feces were collected in the delivery room of sows, which were placed in individual crates. The feeding management, immunization procedures, and epidemic prevention were according to the regular management of pig farms. PBd was conserved in the China Center for Type Culture Collection (CCTCC No. M20221529, Wuhan, Hubei, China).

PBd was cultured in 50 mL of Gifu Anaerobic medium (GAM, No. HB8518-1, Qingdao, China), in addition to 0.05 mL of Hemin (5 mg/mL, HY-19424) and 5 uL of 1% vitamin K1 (Coolaber, Cat#PM0610-1-5, Beijing, China) solution for 24 h at 37 °C. The counts of viable probiotic bacteria in the PBd-containing supplements were verified via the cultural method using Columbia Blood Agar Base (No. HB0124, Qingdao, China). ETEC was kindly provided by Prof. Wenkai Ren from South China Agricultural University (Guangzhou, China) and cultured in LB medium (No. HB0128, Qingdao, China) for 12 h at 37 °C.

**Figure 1:**
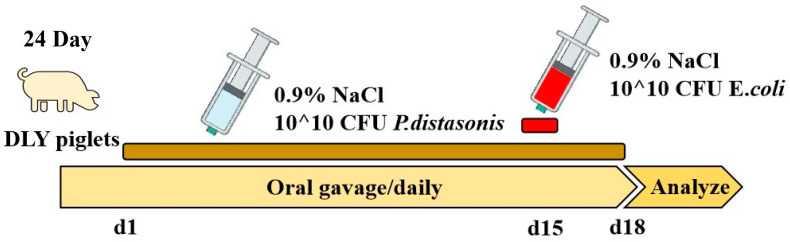
Experimental design diagram. From 1 to 18 d, Control group and ETEC group received a standard diet and daily gavage of 10 mL of sterile saline; PBd + ETEC group received a standard diet and gavage of 10^10^ CFU (10 mL) of *Parabacteroides distasonis* (PBd) every day. At d 15, all groups except the Control group received a gastric infusion of 10^10^ CFU (10 mL) of ETEC, and the Control group received a gastric infusion of sterile saline using the same method.

A total of twenty-two 24-day-old crossbred male piglets [Duroc × (Landrace × Yorkshire)] were selected from the Liuyang Qingquan Cooperative, Hunan, China, and randomly divided into three groups with similar body weights: the Control group (*n* = 7), ETEC group (*n* = 7), and PBd + ETEC (*n* = 8). From 1 to 18 d, the Control group and ETEC group received a standard diet and daily gavage of 10 mL of sterile saline, and the PBd + ETEC group received a standard diet and gavage of 10^10^ CFU (10 mL) PBd every day. At d 15, all groups except the Control group received a gastric infusion of 10^10^ CFU (10 mL) of ETEC, and the Control group received a gastric infusion of sterile saline using the same method (Figure 1). All piglets were housed (2 m × 1.2 m) and fed individually. No antibiotics were given to the animals throughout the trial for prophylactic or therapeutic reasons. All piglets had free access to feeding and drinking water. Room temperature was maintained at approximately 27–30 °C, and the humidity was controlled between 50 and 60%. The formula of the basic diet followed the NRC (2012) for swine nutrition. The basic formula and nutritional levels are presented in Table S1. Oral administration lasted 17 days from the age of 24 to 40 days. Early in the morning of d 18, piglets were sacrificed by exsanguination after electrical stunning, and serum and intestine (digesta and tissue samples) were collected and stored at −80 °C for further analysis. Visceral organs (liver and spleen) were also removed after sacrifice. Then, 2–3 cm segments of intestinal tissue (including duodenum, jejunum, and colon) were preserved in 10% formalin for subsequent morphology analysis.

### 2.2. Growth Performance and Relative Organ Weight

Each piglet was weighed on d 1 and d 18 of the experiment. The daily feed intake of each piglet was recorded. The values of the average daily feed intake and average daily gain were calculated. The liver and spleen were weighed, and the relative weight of each organ was calculated as follows: organ weight/final live body weight.

### 2.3. Histopathology

For histologic analysis, the duodenum, jejunum, and colon (mid-section) tissues were fixed in 10% formalin for 24 h at room temperature, embedded in paraffin, and sectioned at a thickness of 3 mm for staining. Sections were analyzed under a microscope (BX53, Olympus, Tokyo, Japan) at magnifications of 40× after hematoxylin and eosin staining, as previously described [17]. The measurement of villi height and crypt depth was performed using ImageJ-1.52a software. Three different sections per piglet and intestinal sections were evaluated. Villus heights/crypt depths were randomly measured at five different locations in each section. Histopathologic injury scores were determined according to Feng’s publication (0, normal mucosal villi; 1, subepithelial Gruenha-gen’s space (oedema); 2, extension of the subepithelial space with moderate lifting of epithelial layer from the lamina propria; 3, massive epithelial lifting down the sides of villi; 4, denuded villi with lamina propria and dilated capillaries exposed; 5, digestion and disintegration of lamina propria) [18].

### 2.4. Real-Time Reverse Transcription PCR

The relative mRNA levels of Bax, Caspase3, Caspase8, Caspase9, Nrf2, SOD, GSH-PX, mTOR, Beclin1, Myd88, P65, TNF-α, IL-1β, IL-6, IL-8, and IL-10 were determined by real-time quantitative PCR. Total RNA of jejunum tissues was prepared using the RNeasy Kit (Qiagen, Hilden, Germany). cDNA was synthesized using 1 μg aliquot of total RNA according to the manufacturer’s instructions (AG11711, Accurate Biotechnology Co., Ltd., Changsha, China). A 7500 Fast Real-Time PCR system (Applied Biosystems, Foster City, CA, USA) was used for quantitative PCR analysis. Real-time PCR was performed at a total reaction volume of 10 μL, including 5 μL of SYBR Green Premix (AG11701, Accurate Biotechnology Co., Ltd., Changsha, China), 4 μL of 20-fold diluted cDNA, 0.5 μL of forward primers (10 μM) and 0.5 μL of reverse primers (10 μM). The primers (Tsingke Biotechnology Co., Ltd., Bejing, China) used are listed in Supplementary Table S2. The relative mRNA expression level was calculated using the 2^−ΔΔCT^ method, and the results were normalized to β-actin housekeeping genes.

### 2.5. Serum Inflammatory Cytokine

Blood samples were centrifuged for 90 s at 15,000× *g*, and serum aliquots were snap-frozen until further analysis. The quantification of cytokine levels (including IL-4, IL-6, IL-8, IL-10, IL-1β, and TNF-α) in serum was assessed using a commercially available porcine cytokine multiplex immunoassay kit (RayBiotech, Norcross, GA, USA), and the concentration of cytokines was calculated by QAP-CYT-1-SW software (RsyBiotech, Peachtree Corners, GA, USA).

### 2.6. SCFAs

The concentrations of short-chain fatty acids (SCFAs; acetate, propionate, butyrate, and valerate) in colonic chyme were analyzed using the gas chromatographic method. Briefly, as previously reported [19], 1.0 g of colonic chyme was first fully homogenized with 1.5 mL of deionized water. The above fecal homogenate was centrifuged at 15,000× *g* for 10 min at 4 °C. The samples were acidified with 25% metaphosphoric acid at a ratio of 1:5 for 30 min on ice. Samples were injected into a gas chromatographic 8890 series gas chromatograph (Agilent, Santa Clara, CA, USA) for detection.

### 2.7. Statistical Analysis

The data were analyzed using SPSS 26.0 statistical software (ver. 26.0 for Windows, SPSS Inc., Chicago, IL, USA). Firstly, the Shapiro–Wilk method was used to determine whether the data conformed to the normal distribution, and one-way ANOVA and Duncan’s method were used to compare the differences between the groups. All data were expressed as means with their standard errors. A *p*-value < 0.05 was considered statistically significant, and a 0.05 ≤ *p*-value < 0.1 was considered a statistical trend.

## 3. Results

### 3.1. Growth Performance and Relative Organ Weight

As presented in Table 1, compared to the Control group, the ETEC challenge significantly decreased final body weight (FBW, *p* = 0.010), average daily gain (ADG, *p* = 0.010), and average daily feed intake (ADFI, *p* = 0.038) (*p* < 0.05). When compared to the ETEC group, oral administration of PBd significantly increased FBW and ADG in ETEC-challenged piglets (*p* = 0.019, Table 1). However, the PBd + ETEC group did not show a significant difference in ADFI compared to the ETEC group (*p* = 0.058, Table 1). Additionally, no differences were identified in the feed/gain (F/G) ratio among the three groups (*p* > 0.05, Table 1).

Furthermore, the ETEC challenge significantly increased anus temperature (*p* = 0.023), whereas oral administration of PBd showed a tendency to decrease it (*p* < 0.1, Table 1). Compared with the Control group, the ETEC challenge significantly increased the spleen index (*p* = 0.028, Table 1), but there was no significant difference in the spleen index between the PBd + ETEC and ETEC groups (*p* > 0.05, Table 1). No differences were identified in the liver index among the three groups (*p* > 0.05, Table 1).

### 3.2. Intestinal Histomorphology

**Figure 2:**
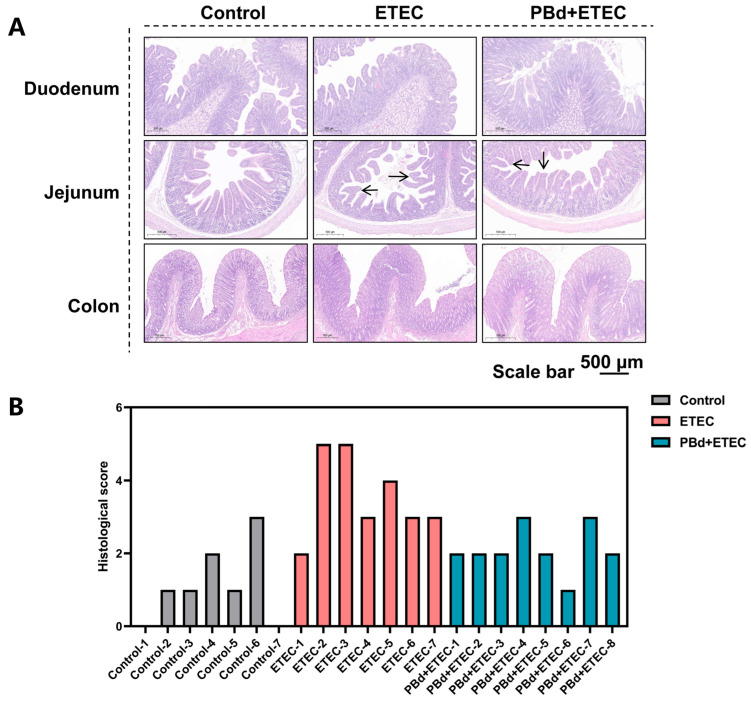
Effects of *P. distasonis* on intestinal morphology in ETEC-challenged piglets. (**A**) Histology image depicting morphometric measurements for the villus height and the crypt depth from intestinal tissue section (hematoxylin and eosin stained) of piglet (scale bar, 500 µm). (**B**) The histological score of each piglet (*n* = 7/8). Arrows indicate intestinal villi.

In the duodenum, the ETEC group significantly increased crypt depth (*p* = 0.017) and reduced villus height (*p* = 0.001) and the villus height/crypt depth (V/C, *p* = 0.001) ratio compared to the Control group. Oral administration of PBd significantly reversed these changes in ETEC-challenged piglets (Figure 2A and Table 2). In the jejunum, the ETEC group significantly reduced villus height (*p* = 0.001) and the V/C (*p* = 0.001) ratio compared to the Control group, whereas oral administration of PBd significantly increased these parameters in ETEC-challenged piglets (*p* < 0.001, Figure 2A and Table 2). The histological score shows that the ETEC challenge caused jejunum injury in piglets, and PBd could reduce jejunum injury caused by ETEC infection compared with the Control group (*p* < 0.05, Figure 2B and Table 2). Additionally, there were no significant differences in crypt depth among the three groups in both the jejunum and colon (*p* > 0.05, Figure 2 and Table 2).

### 3.3. Expression of Apoptosis-Related Genes

**Figure 3:**
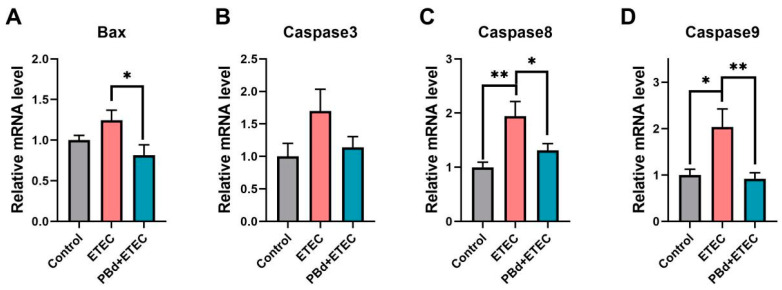
Effects of *P. distasonis* on intestinal apoptosis-related genes in ETEC-challenged piglets. The mRNA expression of Bax (**A**), Caspase3 (**B**), Caspase8 (**C**), and Caspase9 (**D**) in jejunum of piglets. Data are presented as the means ± SEM (*n* = 6).“*” 0.01 ≤ *p* ≤ 0.05; “**” 0.001 < *p* ≤ 0.01.

As shown in Figure 3, the ETEC challenge significantly increased the mRNA expression of *Caspase 8* (*p* = 0.02) and *Caspase 9* (*p* = 0.011) compared to the Control group. Oral administration of PBd significantly decreased the mRNA expression of *Bax* (*p* = 0.018), *Caspase 8* (*p* = 0.017), and *Caspase 9* (*p* = 0.004) in ETEC-challenged piglets (Figure 3A,C,D). However, there were no significant differences in the mRNA expression of *Caspase 3* among the three groups (*p* > 0.05, Figure 3B).

### 3.4. Expression of Antioxidant-Related Genes

**Figure 4:**
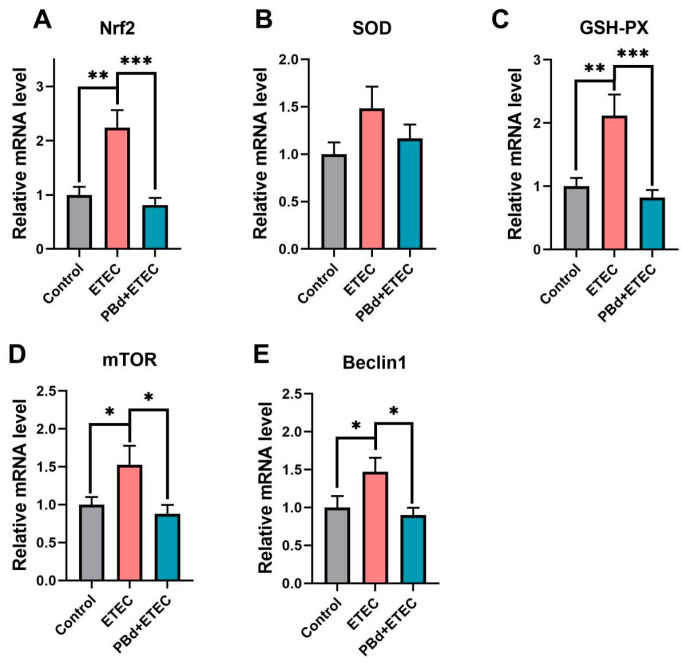
Effects of *P. distasonis* on the antioxidant-related genes in ETEC-challenged piglets. The mRNA expression of Nrf2 (**A**), SOD (**B**), GSH-PX (**C**), mTOR (**D**), and Beclin1 (**E**) in jejunum of piglets. Data are presented as the means ± SEM (*n* = 6). “*” 0.01 ≤ *p* ≤ 0.05; “**” 0.001 < *p* ≤ 0.01; “***” *p* ≤ 0.001.

As presented in Figure 4, the ETEC challenge significantly increased the mRNA expression of *Nrf2* (*p* = 0.002), *GSH-PX* (*p* = 0.003), *mTOR* (*p* = 0.042), and *Beclin1* (*p* = 0.041) compared to the Control group. Oral administration of PBd significantly decreased the mRNA expression of these genes in ETEC-challenged piglets (*p* < 0.05, Figure 4A,C,E. However, there were no significant differences in the mRNA expression of *SOD* among the three groups (*p* > 0.05, Figure 4B).

### 3.5. Inflammatory Biomarkers

**Figure 5:**
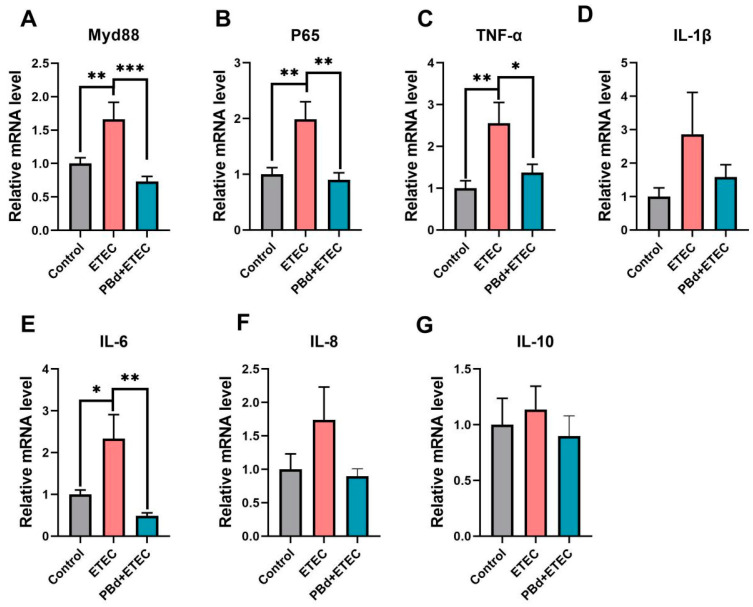
Effects of *P. distasonis* on inflammatory expression in ETEC-challenged piglets. The mRNA expression of Myd88 (**A**), P65 (**B**), TNF-α (**C**), IL-1β (**D**), IL-6 (**E**), IL-8 (**F**), and IL-10 (**G**) in jejunum of piglets. Data are presented as the means ± SEM (*n* = 6). “*” 0.01 ≤ *p* ≤ 0.05; “**” 0.001 < *p* ≤ 0.01; “***” *p* ≤ 0.001.

In the serum, the ETEC challenge significantly increased IL-6 (*p* = 0.021) and IL-10 (*p* = 0.029) levels compared to the Control group, while PBd + ETEC significantly reduced IL-6 levels (*p* < 0.05, Table 3). Compared to the PBd + ETEC group, no differences in the levels of IL-10 were detected between the CON group and the ETEC group (*p* = 0.089, Table 3). There were no significant differences in TNF-α, IL-1β, IL-4, and IL-8 among the three groups (*p* > 0.05, Table 3). In the jejunum, the ETEC challenge significantly increased the mRNA expression of *Myd88* (*p* = 0.005), *P65* (*p* = 0.005), *TNF-α* (*p* = 0.006), and *IL-6* (*p* = 0.024) levels compared to the Control group, while PBd + ETEC significantly reduced the mRNA expression of *Myd88* (*p* = 0.001), *P65* (*p* = 0.001), *TNF-α* (*p* = 0.015), and *IL-6* (*p* < 0.05, Figure 5A,C,F). However, there were no significant differences in the mRNA expression of *IL-1β*, *IL-8*, and *IL-10* among the three groups (*p* > 0.05, Figure 5B,D,E).

### 3.6. SCFAs

**Figure 6:**
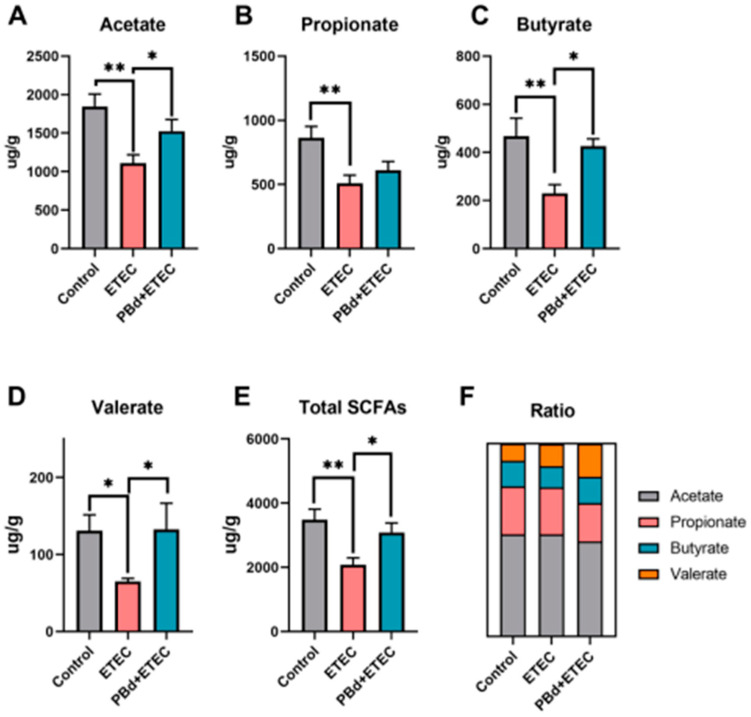
Effects of *P. distasonis* on colonic SCFAs in ETEC-challenged piglets. The SCFAs composition in colonic chyme: acetate (**A**), propionate (**B**), butyrate (**C**), valerate (**D**), total SCFAs (**E**), and the relative content of each SCFA in different groups, obtained by dividing a single SCFA per sample by the total SCFA (**F**). Data are presented as the means ± SEM (*n* = 6). “*” 0.01 ≤ *p* ≤ 0.05; “**” 0.001 < *p* ≤ 0.01.

Furthermore, we measured the levels of SCFAs in the colonic chyme (Figure 6). The results show that the ETEC challenge significantly reduced the content of acetate (*p* = 0.002), propionate (*p* = 0.003), butyrate (*p* = 0.007), valerate (*p* = 0.050), and total SCFAs (*p* = 0.002) compared to the Control group (Figure 6A–E). Oral administration of PBd significantly increased the content of acetate (*p* = 0.050), butyrate (*p* = 0.043), valerate (*p* = 0.049), and total SCFAs (*p* = 0.029) in ETEC-challenged piglets (Figure 6A,C–E). The ratio of acetate, propionate, butyrate, and valerate in total SCFAs was calculated and is shown in Figure 6F, suggesting that oral administration of PBd had a higher proportion of butyrate and valerate in the colonic chyme compared to the ETEC group (*p* < 0.05).

## 4. Discussion

Post-weaning piglets often experience reduced appetite and feed intake due to weaning stress, significantly impacting their growth and development [20,21,22]. Studies have demonstrated that probiotics can maintain gut microbiota balance, enhance nutrient digestibility, and consequently promote growth [23,24]. In this study, we found that oral administration of PBd significantly increased the ADG of piglets and reduced the weight loss caused by the ETEC challenge.

Previous studies have shown that ETEC can colonize and secrete exotoxins in the host small intestine, damaging intestinal health [25]. Xu et al. reported that the jejunum is the main part of injured intestines in the ETEC challenge [26]. Similarly, Ren et al. analyzed the jejunal protein spectrum and found that ETEC infection could promote the activation of NF-κB and MAPK pathways, and the metabolic process and binding function of jejunal tissue were most seriously affected in ETEC-induced diarrhea [27]. The small intestine is the main place to absorb nutrients [1]; indicators, such as villus height (VH), crypt depth (CD), and the villus height/crypt depth ratio (V/C) in the small intestine are vital measures of digestive and absorptive capacity [1]. Numerous studies have shown that probiotics can increase the length of small intestinal villi and reduce crypt depth [28,29,30]. Moreover, weaning piglets undergo physiological changes in the intestine, such as villus shortening and damage, making them susceptible to pathogenic infections. A previous study reported that the lipopolysaccharide stimulation of weaned piglets could lead to necrosis and reduced villus height in the jejunum [31]. As expected, our results show that the ETEC challenge resulted in the destruction of the intestinal integrity of piglets and led to intestinal injury, while oral administration of PBd significantly reversed these changes. This finding is similar to the study by Wu T et al., who found that *Lactobacillus rhamnosus Lb1* alleviates the intestinal injury caused by ETEC and has beneficial effects on intestinal integrity [32]. In conclusion, PBd can improve the intestinal morphology of weaning piglets, thereby enhancing their growth performance.

The structural integrity of intestinal mucosal cells is the morphological basis for maintaining mucosal function [33]. Under normal circumstances, intestinal mucosal cells constantly renew themselves, and apoptosis plays a role in cell renewal and repair. The balance between proliferation and apoptosis of intestinal mucosal cells is fundamental to maintaining mucosal integrity and function. Bax and Caspase families are key pro-apoptotic genes that play crucial roles in the process of apoptosis [34,35]. Bax, a member of the Bcl-2 family, promotes mitochondrial membrane permeability by forming heterodimers with Bcl-2, leading to the release of cytochrome c and initiating apoptosis [34]. The Caspase family includes a series of proteases, such as Caspase-3, Caspase-8, and Caspase-9, which execute apoptosis through a cascade reaction, ultimately disrupting cell structure and function [35]. The coordinated action of Bax and Caspases ensures that cells can effectively initiate apoptosis when damaged or no longer needed, maintaining organism stability and health [36]. In this study, the ETEC challenge significantly increased the expression of apoptosis-related genes, such as Caspase-8 and Caspase-9, which is similar to Xia’s results [37]. Previous studies have shown that Escherichia coli shiga can activate Caspase-9 in HRT-18 and IEC-18 cell lines [38]. These data indicate that the ETEC challenge caused apoptosis in the piglet jejunum, whereas oral administration of PBd significantly suppressed the expression of apoptosis-related genes, such as Bax, Caspase-8, and Caspase-9, in the jejunal tissue of ETEC-challenged piglets. These results suggest that oral administration of PBd reduces ETEC-induced intestinal injury by improving the imbalance between cell apoptosis and cell regeneration in ETEC-challenged piglets.

Oxidative stress is a major factor affecting the growth performance of weaning piglets, as oxygen-free radicals generated by metabolism can cause cell damage and induce various diseases [39,40]. The body’s antioxidant capacity is closely related to animal health, and many studies have shown that probiotic preparations can enhance the antioxidant capacity of animals and improve health status [41,42]. Nrf2 is a key nuclear transcription factor that regulates oxidative stress by binding to antioxidant response elements and inducing the expression of antioxidant enzyme-related genes [43]. Previous studies have shown that the concentration of Nrf2 in cancer tissues and organs increases steadily [44]. Additionally, mTOR regulates cellular antioxidant mechanisms to maintain redox balance, thus reducing oxidative stress-induced cellular damage [45]. Beclin1 is an important regulatory factor in the autophagy process, which maintains cellular homeostasis by clearing damaged organelles and proteins [46]. Under oxidative stress, Beclin1-mediated autophagy is crucial for removing oxidative damage and maintaining cell function. Our results show that the ETEC challenge upregulated the mRNA expression of Nrf2 and GSH-Px, which is consistent with Wen’s research results, and PBd intervention reversed this change [47]. In addition, PBd also significantly downregulated the expression of mTOR and Beclin1. This indicates that PBd from Ningxiang pigs enhances antioxidant capacity and reduces the damage of ETEC treatment to tissues.

Post-weaning, the atrophy of intestinal villi in piglets can trigger inflammatory responses. Myd88 and p65 play crucial roles in inflammatory responses, particularly in regulating the expression of inflammatory cytokines, such as IL-1β, IL-6, IL-10, and TNF-α [48]. Myd88 is a key adaptor protein in the Toll-like receptor (TLR) signaling pathway, mediating the activation of the TLR signaling pathway to activate NF-κB and induce inflammatory responses [49]. NF-κB p65 is an important component of the NF-κB pathway. Upon TLR activation via Myd88, NF-κB p65 translocates to the nucleus and promotes the expression of inflammatory genes. Studies have shown that the activation of these pathways in weaning piglets leads to a significant increase in inflammatory cytokines, such as IL-1β, IL-6, IL-10, and TNF-α [50,51]. Liu et al. found that PBd may improve the inflammatory response of T2D rats by regulating the TLR4/NF-κB pathway [52]. In addition, a previous study showed that PBd is anti-inflammatory by reducing the signal pathways of TLR4, Myd88, and Akt and stimulates apoptosis [53], which is partly consistent with our results. Our study finds that oral administration of PBd can reduce the levels of inflammatory cytokines in weaning piglets by modulating the TLR-Myd88-NF-κB signaling pathway, thereby enhancing their anti-inflammatory capacity. Significantly, our results show the different expression of TNF-α, IL-6, and IL-8 in serum and jejunum tissue. This may be related to tissue specificity. The serum shows a high degree of variability and captures immediate responses, while gut tissue is more stable and responds to chronic or sustained changes, which may be the reason for the differences in our results. This study indicates that PBd from Ningxiang pigs can inhibit the production of pro-inflammatory cytokines, thereby helping to maintain intestinal health and reduce weaning-induced intestinal inflammation.

One of the mechanisms by which probiotics inhibit the proliferation of intestinal pathogens is through the fermentation of carbohydrates in the animal’s gut, producing substantial amounts of SCFAs (acetate, propionate, and butyrate) [54]. SCFAs have been identified as regulators of intestinal metabolism, proliferation, and differentiation and can promote intestinal homeostasis by exerting anti-inflammatory effects [55]. Butyrate, in particular, is a crucial energy source for intestinal epithelial cells, improving host gut microbiota dysbiosis and significantly increasing the relative abundance of beneficial bacteria [56]. Additionally, valerate can enhance intestinal barrier function at physiological concentrations [57]. In this study, oral administration of PBd significantly increased the levels of acetate, butyrate, and valerate in the colon of ETEC-challenged piglets. Our result shows that PBd derived from Ningxiang pigs can help maintain gut health and reduce weaning-induced intestinal inflammation by promoting the production of SCFAs in the host.

## 5. Conclusions

**Figure 7:**
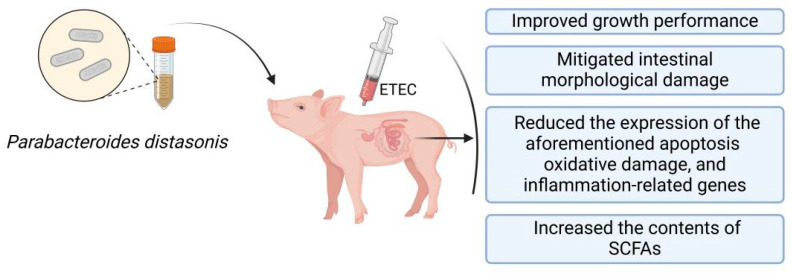
Schematic diagram of how PBd can improve intestinal function and attenuate ETEC-induced intestinal damage in piglets.

Overall, PBd derived from Ningxiang pigs can reduce intestinal cell apoptosis levels in ETEC-challenged piglets, enhance their intestinal antioxidant capacity and anti-inflammatory levels, improve intestinal morphology, and increase the contents of SCFAs in the colonic chyme, thereby promoting the growth of weaned piglets (Figure 7). It is necessary to study further the potential mechanisms by which PBd improves intestinal health in ETEC-challenged piglets and clarify the interaction between PBd derived from Ningxiang pigs and ETEC. Although this study has some limitations, these results still provide an effective method for preventing and treating intestinal injury caused by ETEC infection.

Future research should focus on the following aspects: 1. Delve deeper into the regulatory mechanisms of Ningxiang pig-derived PBd on ETEC infection, identifying the specific molecular and cellular pathways involved; 2. Investigate the dynamic interactions between PBd and ETEC within the intestinal microecological environment, analyzing their impact on gut microbiota structure and function and 3. Explore the synergistic effects of PBd with other gut health interventions to develop more effective integrated prevention and treatment strategies.

## Figures and Tables

**Table 1:** Effects of *P. distasonis* on the growth performance and relative organ weight of ETEC-challenged piglets.

Items	Control	ETEC	PBd + ETEC
IBW, kg	6.22 ± 0.11	6.40 ± 0.09	6.30 ± 0.12
FBW, kg	11.70 ± 0.52 ^a^	9.87 ± 0.51 ^b^	11.43 ± 0.23 ^a^
ADG, kg/d	0.68 ± 0.031 ^a^	0.58 ± 0.03 ^b^	0.67 ± 0.013 ^a^
ADFI, kg/d	0.43 ± 0.027 ^a^	0.34 ± 0.02 ^b^	0.42 ± 0.02 ^ab^
F/G, kg/kg	0.64 ± 0.05	0.60 ± 0.05	0.63 ± 0.04
Anus temperature, °C	39.36 ± 0.54 ^b^	40.56 ± 0.22 ^a^	39.80 ± 0.10 ^ab^
Liver, g/kg	2.58 ± 0.08	2.81 ± 0.14	2.66 ± 0.10
Spleen, g/kg	0.19 ± 0.01 ^b^	0.27 ± 0.02 ^a^	0.23 ± 0.02 ^ab^

Note: ^a,b^ In the same row, different superscript letters indicate significant differences (*p* < 0.05), and the same superscript letters indicate no significant difference (*p* > 0.05). Data are presented as the means ± SEM (*n* = 7–8). IBW, initial body weight. FBW, final body weight. ADG, average daily gain. ADFI, average daily feed intake. F/G, average daily feed intake to average daily gain.

**Table 2:** Effects of *P. distasonis* on the intestinal histomorphology of ETEC-challenged piglets.

Items	Control	ETEC	PBd + ETEC
**Duodenum**			
Villus height, µm	371.49 ± 90.54 ^a^	240.26 ± 51.49 ^b^	394.63 ± 30.25 ^a^
Crypt depth, µm	307.79 ± 61.00 ^b^	389.90 ± 66.28 ^a^	289.11 ± 49.10 ^b^
V/C	1.26 ± 0.43 ^a^	0.63 ± 0.19 ^b^	1.40 ± 0.29 ^a^
**Jejunum**			
Villus height, µm	531.15 ± 64.84 ^a^	204.25 ± 56.75 ^b^	477.49 ± 73.06 ^a^
Crypt depth, µm	195.53 ± 36.67	241.98 ± 64.55	224.41 ± 21.99
V/C	2.77 ± 0.42 ^a^	0.86 ± 0.21 ^c^	2.13 ± 0.27 ^b^
Histological score	0.86 ± 0.26 ^c^	3.57 ± 0.43 ^a^	1.87 ± 0.30 ^b^
**Colon**			
Crypt depth, µm	464.06 ± 27.62	579.28 ± 133.37	515.48 ± 150.61

Note: ^a,b,c^ In the same row, different superscript letters indicate significant differences (*p* < 0.05), and the same superscript letters indicate no significant difference (*p* > 0.05). Data are presented as the means ± SEM (*n* = 7–8). V/C, villus height to crypt depth.

**Table 3:** Effects of *P. distasonis* on the inflammatory cytokine levels in the serum of ETEC-challenged piglets.

Items	Control	ETEC	PBd + ETEC
TNF-α, pg/mL	208.05 ± 89.34	476.97 ± 135.02	225.46 ± 41.37
IL-1β, pg/mL	479.44 ± 91.27	730.56 ± 149.28	434.22 ± 84.70
IL-4, pg/mL	293.38 ± 96.70	1191.60 ± 514.37	454.41 ± 94.09
IL-6, pg/mL	157.50 ± 44.16 ^b^	653.31 ± 215.21 ^a^	201.53 ± 60.29 ^b^
IL-8, pg/mL	35.15 ± 3.84	49.72 ± 9.78	55.85 ± 15.05
IL-10, pg/mL	837.04 ± 243.38 ^b^	1864.81 ± 447.15 ^a^	1180.25 ± 212.88 ^ab^

Note: ^a,b^ In the same row, different superscript letters indicate significant differences (*p* < 0.05), and the same superscript letters indicate no significant difference (*p* > 0.05). Data are presented as the means ± SEM (*n* = 6).

## Data Availability

Data are contained within the article and Supplementary Materials.

## Supplementary Materials

The following supporting information can be downloaded at: https://www.mdpi.com/article/10.3390/ani14152156/s1, Table S1: Basic diet composition and nutritional component (%, as-fed basis); Table S2: Primers used for gene expression analysis by real-time.
