# Supplementary material for: Ningxiang Pig-Derived Parabacteroides distasonis HNAU0205 Alleviates ETEC-Induced Intestinal Apoptosis, Oxidative Damage, and Inflammation in Piglets
Source: Animals (Basel). 2024 Jul 24;14(15):2156. doi: 10.3390/ani14152156 (PMC11310999; doi:10.3390/ani14152156)
Supplement: Supplementary file 1 [file animals-14-02156-s001.zip › animals-3088117-supplementary.pdf]

Tables

Table S1. Basic diet composition and nutritional component (%, as-fed basis).

Item	Contents
**Ingredients**	
Corn	42.02
Soybean meal	20.00
Extruded full-fat soybean	12.00
Soy protein concentrate	4.00
Fermented cottonseed meal	0.00
Whey powder	8.00
Soy oil	4.61
Sucrose	5.38
Dicalcium phosphate	1.26
Limestone	0.99
Salt	0.30
Lysine	0.41
Methionine	0.13
Threonine	0.13
Tryptophan	0.02
Chromic oxide	0.25
Vitamin-mineral premix[1], no antibiotic	0.50
**Total**	100.00
**Nutrient levels**[2]	
Metabolized energy, kcal/kg	3.40
Digestible energy, Mcal/kg	3.47
Dry matter	87.60
Crude protein	24.50
Total calcium	0.63
Total phosphorus	0.65
Total lysine	1.51
Total threonine	0.94
Total tryptophan	0.40
Total methionine + cystine	0.86

[1] The components and contents of the premix providing nutrients for per kg feed are as follows: Vitamin A, 12,000 IU; Vitamin D3, 2500 IU; Vitamin E, 30 IU; Vitamin K3, 30 mg; Vitamin B12, 12 micrograms; Riboflavin, 4 mg; Pantothenic acid, 15 mg; Niacin, 40 mg; Choline chloride, 400 mg; Folic acid, 0.7 mg; Vitamin B1, 1.5 mg; Vitamin B6, 3 mg; Biotin, 0.1 mg; Manganese, 40 mg; Iron, 90 mg; Zinc, 100 mg; Copper, 8.8 mg; Iodine, 0.35 mg; Selenium, 0.3 mg.

Table S2. Primers used for gene expression analysis by real-time.

Gene	Primer sequence (5’-3’)
Myd88	F: GTGCCGTCGGATGGTAGTG
	R: TCTGGAAGTCACATTCCTTGCTT
P65	F:AGCCATTGACGTGATCCAGG
	R:CGAAATCGTGGGGCACTTTG
TNF-α	F: ATCGGCCCCCAGAAGGAAGAG
	R:GATGGCAGAGAGGAGGTTGAC
IL-1β	F: AACGTGCAGTCTATGGAGT
	R: GAACACCACTTCTCTCTTCA
IL-6	F: CTGCTTCTGGTGATGGCTACTG
	R: GGCATCACCTTTGGCATCTT
IL-8	F: AGTTTTCCTGCTTTCTGCAGCT
	R: TGGCATCGAAGTTCTGCACT
IL-10	F: ACCAGATGGGCGACTTGTTG
	R: CACTCTCTGCCTTCGGCATT
Nrf2	F: GAGCGGGAAGAAAGGCAGAA
	R: CGTTGCACATATTTGAGGTCCA
SOD	F: TCCATGTCCATCAGTTTGGA
	R: CTGCCCAAGTCATCTGGTTT
GSH-PX	F: GGCACAACGGTGCGGGACTA
	R: AGGCGAAGAGCGGGTGAGCA
mTOR	F: AGCCTTCGTCTATGACCCCT
	R: CGGCAGAGTAGGAATCCGTC
Beclin1	F: TCCATTACTTGCCACAGCCC
	R: CCCGATCAGAGTGAAGCTGT
BaX	F: CTGACGGCAACTTCAACTGG
	R: CGTCCCAAAGTAGGAGAGGA
Caspase3	F: CGTGCTTCTAAGCCATGGTG
	R: GTCCCACTGTCCGTCTCAAT
Caspase8	F: AGACAAGGGCATCATCTACGG
	R: GGGTTTACCAAGAAGGGAAGG
Caspase9	F: AATGCCGATTTGGCTTACGT
	R: CATTTGCTTGGCAGTCAGGTT
β-actin	F: CTGCGGCATCCACGAAACT
	R: AGGGCCGTGATCTCCTTCTG
